# Utilization of Tunnel Muck-Derived Recycled Granite Aggregates in Surface-Layer Asphalt Mixtures via Hybridization with Basalt

**DOI:** 10.3390/ma18194611

**Published:** 2025-10-05

**Authors:** Yuqi Zhou, Weiwei Liu, Yanxia Nie, Zongwu Chen

**Affiliations:** 1 China Construction First Group Construction & Development Co., Ltd., Beijing 100102, China; zhouyuqidoc@163.com (Y.Z.); ww9812004@163.com (W.L.); nieyanxia@chinaonebuild.com (Y.N.); 2 School of Materials Science and Engineering, Chang’an University, Xi’an 710061, China; 3 Faculty of Engineering, China University of Geosciences (Wuhan), Wuhan 430074, China

**Keywords:** tunnel muck, recycled granite aggregate, hybrid aggregate, material property, surface-layer asphalt mixture, engineering performance

## Abstract

This study explored the feasibility of utilizing tunnel muck-derived recycled granite aggregates (RGAs) in surface-layer asphalt mixtures via hybrid with basalt aggregates. Firstly, RGAs, including coarse aggregates (RGCAs) and fine aggregates (RGFAs), were prepared using a production method integrated with multi-cleaning technology. Then, the material properties of RGAs and RGA–basalt hybrid aggregates with varying RGA volume proportions were investigated. Finally, asphalt mixtures with these hybrid aggregates were designed and their engineering performance was evaluated. Basalt aggregates and their corresponding asphalt mixture served as the control group. Results suggest that since RGAs are rich in quartz and their SiO_2_ content is as high as 70.88%, they are acidic aggregates. Employing multi-cleaning technology is a guaranteed method of obtaining RGAs with low mud content. The main conventional technical indexes of RGAs and all hybrid aggregates with 40–70% RGA volume proportions meet the requirements of Chinese technical specifications. Asphalt mixtures incorporating RGAs exhibit slightly higher voids in the mineral aggregates (VMAs) than the control group, indicating that RGAs modify the interlocking skeleton and contact states of aggregates. Blending RGAs with basalt to form hybrid aggregates is an effective way to achieve full-gradation utilization of tunnel muck-derived RGAs in the surface-layer asphalt mixtures. Without additional enhancement measures, a 40% RGA volume proportion in hybrid aggregates is recommended. For a higher RGA recycling rate, combining RGAs with cement is advised, maintaining 70% RGA volume proportion and 50% cement content of total filler volume. When external basalt aggregates are transported over a distance of 50–200 km, applying these schemes to local asphalt pavement surface layers can achieve at least 26.56% aggregate cost savings.

## 1. Introduction

Tunnels play an indispensable role in the development of high-speed highways and railways [1], especially in mountainous regions. They effectively overcome topographical barriers like steep slopes, deep valleys, and complex terrain, significantly shortening the length of transportation routes, and reducing travel time. China is a mountainous country. To promote the development of mountainous regions and facilitate daily travel for local people, the construction of high-speed highways and railways in mountainous regions is an ongoing task in China. As early as 2015, Wang stated that there were 7000 km of mountainous tunnels under construction and about 10,000 km of mountainous tunnels were ready for construction [2]. As a result, excavating numerous tunnels produces a large amount of tunnel muck [3]. The efficient utilization of this waste is a big challenge that is currently being faced.

Asphalt pavement is widely used worldwide due to its multiple functions such as good smoothness, ease of maintenance, ability to ensure comfortable driving, producing a low level of noise, etc. [4]. In China, due to the prevalence of heavy traffic, about 90% of high-grade asphalt pavement adopts a combined structure of asphalt layer and semi-rigid base layer [5], which can provide better load-bearing capacity while also reducing construction costs. The asphalt layer usually consists of three different layers: a surface layer, an intermediate layer, and a bottom layer [6]. As the surface layer directly interacts with the vehicle tires and multiple environmental factors, aggregates with high wear resistance and anti-skid capability are required to maintain the excellent performance of the pavement surface. For example, wear-resistant and anti-skid basalt aggregates are widely used in the surface-layer asphalt mixtures in China. According to the latest statistics from the Chinese Ministry of Transport, the total mileage of highways in China reached 5.4904 million kilometers by the end of 2024, which is an increase of about 5.6% over the past five years. It indicates that the construction and maintenance of highways in China is still a heavy burden. However, with the continuous construction of asphalt pavement, the shortage of high-quality aggregates is becoming a serious issue. For example, the supply distance of basalt aggregates in Hubei Province, China has exceeded one hundred kilometers, which creates a significant challenge for asphalt pavement construction, especially in mountainous regions. Therefore, developing suitable alternative materials is an urgent necessity [7]. Recycling certain waste materials in pavement is a promising way to ensure the sustainable development of traffic roads, and there have been several successful examples of this [8,9,10,11,12,13,14,15]. The use of steel slag [8,9,10], Reclaimed Asphalt Pavement (RAP) [11,12], waste concrete [13,14], etc., is common in asphalt mixtures. In this context, if tunnel muck can be locally used as aggregates for surface-layer asphalt mixtures, it will create a win–win situation, fully utilizing the solid waste generated from tunnel construction while reducing the excavation amount and transportation cost of high-quality aggregates.

Globally, although there have been some studies on the recycling of tunnel muck in civil engineering, such as using it as aggregates for road construction materials [16,17], as granular material for road subgrade and sub-base [3,18], etc., research on its use in asphalt pavement is rare. This is primarily since asphalt mixtures impose stringent requirements on aggregate properties. The high mud and impurity content in tunnel muck poses challenges for obtaining high-quality recycled aggregates. In addition, particularly in China, a significant number of mountainous tunnels traverse acidic granite strata. Acidic aggregates exhibit poor bonding property with asphalt [19,20,21], which ultimately influences the engineering performance of the asphalt mixture. According to previous research, the most effective method to reduce the adverse effects of acidic aggregates on the performance of asphalt mixtures is to blend acidic coarse aggregates with high-quality fine aggregates and fillers to form hybrid mineral mixtures [9,21,22,23]. However, this method neglects the utilization of acidic fine aggregates, thereby failing to achieve full-gradation use of acidic aggregates. Therefore, if this method is adopted, it will limit the utilization efficiency of tunnel muck-derived recycled aggregates in asphalt mixtures.

In order to expand the domains and methods in which tunnel muck could be utilized, the feasibility of using tunnel muck-derived recycled granite aggregates in surface-layer asphalt mixtures via hybridization with basalt aggregates was investigated in this research. Based on the above, the key is to prepare clean granite aggregates and develop a method for realizing the full-gradation use of granite aggregates in asphalt mixtures. Correspondingly, the following three aspects of work were conducted:

(1) Proposing a production method for processing clean tunnel muck-derived recycled granite aggregates (RGAs), and further analyzing the material properties of RGAs, including mineral phases, chemical compositions, conventional technical properties, and their adhesion performance with asphalt binders. Basalt aggregates served as the control group.
(2) Introducing a new method for simultaneously utilizing recycled granite coarse aggregates (RGCAs) and fine aggregates (RGFAs) to form hybrid aggregates with basalt, further analyzing the conventional technical properties of hybrid aggregates with varying RGA volume proportions, and then designing asphalt mixtures incorporating these hybrid aggregates. The pure basalt aggregate-based asphalt mixture served as the control group.
(3) Investigating the main engineering performance of designed asphalt mixtures, including moisture-induced damage resistance, high-temperature deformation resistance, low-temperature crack resistance and fatigue performance, determining the suitable volume proportions of RGAs in hybrid aggregates, and analyzing the economic benefits based on aggregate costs at suitable RGA volume proportions.

## 2. Materials and Methods

### 2.1. Raw Materials

**Figure 1:**
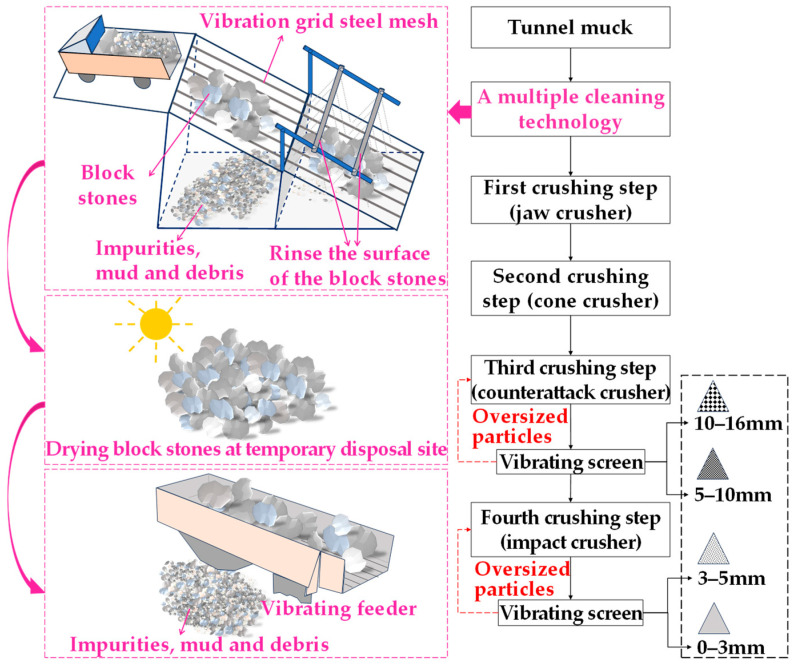
Preparation of RGAs by multi-cleaning and crushing tunnel muck.

Two coarse aggregates (RGCA and basalt), two fine aggregates (RGFA and basalt), and two fillers (limestone powder and cement) were used in this research. RGCAs and RGFAs were prepared by processing tunnel muck using the method illustrated in Figure 1. Tunnel muck was sourced from a high-speed railway tunnel project located at the junction of Hubei Province and Anhui Province in the Chinese Dabie Mountains area. The total length of the tunnel is nearly 15 km, mainly passing through areas with Class II and III surrounding rocks; therefore, the rocks are relatively hard. The drilling and blasting method is used for the construction of the tunnel, which is specifically suitable for hard rock formations like granite. The geological conditions are complex, and some fault fracture zones need to be crossed during construction. Basalt aggregates and limestone powder are obtained from a gravel-processing plant located in Jingshan, Hubei, China. Cement was provided by the Huaxin Cement Co., Ltd., Huangshi, Hubei, China, with a strength grade of 42.5 MPa. In addition, SBS-modified asphalt was used as a binder. It was produced by Hubei Guochuang Hi-Tech Material Co., Ltd., Wuhan, Hubei, China. The conventional technical properties of fillers and Styrene–Butadiene–Styrene (SBS)-modified asphalt were provided by manufacturers, as shown in Table 1 and Table 2. The conventional technical properties of aggregates will be discussed separately in Section 3.1 and Section 3.2.

### 2.2. Experiment Methods

#### 2.2.1. Production Method of RGAs

As shown in Figure 1, the production of RGAs consists of a cleaning stage and a crushing stage. Considering the cleanliness level of tunnel muck is usually low, the impurities, mud, and debris in RGAs will significantly worsen the adhesion performance of aggregates to asphalt. Therefore, it is necessary to deeply clean tunnel muck before crushing it. Before transporting tunnel muck from the excavation site to the temporary disposal site, it is roughly processed, and large debris such as tree branches are manually removed. Then, a multi-cleaning technology is adopted during the cleaning stage to further separate stone blocks from the impurities, mud, and debris mixed in the tunnel muck. The aim is to obtain clean stone blocks for the next crushing stage. Firstly, tunnel muck is subjected to a vibrating grid steel mesh to initially separate large-sized stones from impurities, mud, and debris. The mesh sizes should be flexibly selected according to the actual conditions of the tunnel muck delivered to the site. Subsequently, the residual impurities, mud, and debris on the surfaces of the stone blocks are cleared using a water-flushing device. Finally, the vibration effect applied by the vibrating feeder when feeding stone blocks in the crushing stage removes residual impurities, mud, and debris from the crushed stones again.

Granite possesses high hardness and excellent wear resistance [24]. These features are advantageous when using granite aggregates in surface-layer asphalt mixtures, yet they also pose a challenge in the crushing stage. So, in order to fully crush granite blocks, a four-step crushing technology consisting of coarse, medium, and fine crushing equipment is applied during the crushing stage. The first coarse crushing step uses a jaw crusher. The second medium crushing step utilizes a cone crusher. The third crushing step adopts a counterattack crusher combined with a vibrating screen to separate oversized particles, which are returned to the counterattack crusher for further crushing. After the treatment of this stage, aggregates with two different particle-size ranges (5–10 mm and 10–16 mm) are obtained. The fourth fine crushing step involves an impact crusher, and a vibrating screen is also used to separate oversized particles and obtain aggregates with another two different particle-size ranges (0–3 mm and 3–5 mm). The oversized particles are returned to the impact crusher to be crushed again. In addition, each stage of the crushing process is also equipped with dust-removal devices.

#### 2.2.2. Material Property Evaluation of RGA and Hybrid Aggregates

A D8 Advance X-ray diffraction (XRD) from Bruker, Germany and a Zetium X-ray fluorescence (XRF) from PANalytical, Netherlands were first used to detect the mineral phases and chemical compositions of RGA, respectively. Then, the conventional technical properties of RGCA and basalt coarse aggregates were compared, along with their bonding performance with asphalt. The conventional technical properties of aggregates were tested according to the Chinese standard test methods for aggregates [25]. The bonding performance between aggregates and asphalt was evaluated by the water boiling test, during which aggregate particles with a size of approximately 16 mm were used. The percentage of asphalt stripped from the surface of aggregate particles was determined by Equation (1):

(1) $$S_{p}=\frac{m_{0}-m_{t}}{m_{0}-m}\times 100\%$$

where *S_p_* is the percentage of asphalt stripped (%), *m* is the original dry mass of aggregate particles, and *m*_0_ and *m_t_* refer to the masses of asphalt-coated aggregate particles before and after damage from boiling water for *t* min (g), respectively.

A new method of simultaneously using RGCAs and RGFAs to form hybrid aggregates with basalt aggregates was established after analyzing the current utilization methods of acidic aggregates (see Section 3.2). The conventional technical properties of hybrid aggregates were also tested according to the Chinese standard test methods of aggregates [25].

#### 2.2.3. Design and Performance Evaluation of Asphalt Mixtures

Surface-layer asphalt mixtures with a nominal maximum particle size of 13.2 mm were designed via the Marshall design method, which is widely adopted in China due to its convenience and low cost. The engineering performance of asphalt mixtures was evaluated according to the Chinese standard test methods [26]. The sizes of specimens and main test conditions are shown in Table 3. The main indexes of reflecting each performance were determined with the equations as follows.

(1) Indexes for reflecting the moisture-induced damage resistance

The Marshall test and indirect tensile test were used to evaluate the moisture-induced damage resistance of asphalt mixtures. Marshall stability and indirect tensile strength (ITS) of all specimens were measured by a UTM-130 machine. The retained Marshall stability (RMS) and tensile strength ratio (TSR) indicate the ability of asphalt mixture to resist moisture-induced damage, as shown in Equations (2) and (3).

(2) $$RMS=\frac{MS_{i}}{MS_{0}}\times 100\%$$

(3) $$TSR=\frac{ITS_{j}}{ITS_{0}}\times 100\%$$

where *MS*_0_ and *MS_i_* are the average Marshall stability of specimens before and after hot water damage for *i* h, kN; *ITS*_0_ and *ITS_j_* are the average ITS of specimens before and after freeze–thaw damage for *j* cycles, MPa.

(2) Indexes for reflecting the high-temperature deformation resistance

A rutting test with a wheel-tracking device was adopted to investigate the high-temperature deformation resistance of asphalt mixtures. The steel wheel on the wheel-tracking device moves back and forth repeatedly along the centerline of the surface of plate specimen to form rutting deformation. The dynamic stability shows the ability of asphalt mixtures to resist the high-temperature deformation. Dynamic stability was computed according to Equation (4):

(4) $$DS=\frac{(t_{2}-t_{1})\times s\;}{d_{2}-d_{1}}$$

where *DS* is dynamic stability, pass/mm; *s* is the wheel speed, pass/min; (*d*_2_ − *d*_1_) is the increment of rutting depth from *t*_1_ to *t*_2_ during the stable deformation stage, mm. The single test lasts for 1 h, and (*d*_2_ − *d*_1_) is typically measured from the 45th to 60th minute.

(3) Indexes for reflecting the low-temperature crack resistance

Three-point bending beam test was used to reveal the low-temperature crack resistance of asphalt mixtures. It was carried out also by a UTM-130 machine. The low-temperature performance indexes including the flexural strength, strain, stiffness modulus, and strain energy density of asphalt mixtures were determined by Equations (5)–(8), respectively.

(5) $$\sigma =\frac{3lp}{2bh^{2}}$$

(6) $$\varepsilon =\frac{6hd}{l^{2}}$$

(7) $$S_{f}=\frac{\sigma_{\max}}{\varepsilon_{f}}$$

(8) $$d_{se}=10^{-3}\int_{0}^{\varepsilon_{f}}\sigma d\varepsilon$$

where *σ* is the real-time flexural stress of specimen, MPa; *p* is the real-time force exerted on the specimen during the bending process, kN; *l*, *b* and *h* are the spanning length, width, and height of specimen, respectively, mm; *ε* is the real-time strain that occurs in the specimen, με; *d* is the real-time vertical deflection of specimen, mm; *S_f_* is the flexural stiffness modulus of specimen at failure, MPa; ε*_f_* is the failure strain of specimen, με; *σ*_max_ is the flexural tensile strength of specimen when the strain is ε*_f_*, MPa; *d_se_* is the strain energy density, kJ/m^3^.

(4) Indexes for reflecting the fatigue performance

A four-point bending beam fatigue test was carried out to determine the fatigue performance of asphalt mixtures. Although AASHTO recommends using the *N_f_*_50_ method (loading numbers corresponding to a 50% reduction in initial stiffness modulus) as the criterion for determining the fatigue life of asphalt mixtures, it was not adopted in this research because modified asphalt mixtures show higher toughness and elasticity than unmodified asphalt mixtures. Even if the initial stiffness modulus of modified asphalt mixtures reduces by 50%, it is still far from fatigue failure. In addition, the *N_f_*_50_ method is directly affected by the initial stiffness modulus, which is often difficult to accurately determine, resulting in a large fluctuation in the fatigue life [27]. Therefore, the *N_f_*_NM_ method recommended by ASTM was used in this research. It involves taking the peak of the normalized product of stiffness modulus and loading numbers (Equation (9)) as the fatigue failure point of asphalt mixtures; namely, when reaching its maximum value, the corresponding loading number is defined as fatigue life (*N_f_*_NM_). It can be seen from Equation (9) that although the *N_f_*_NM_ method also involves the initial stiffness modulus, it can be treated as a constant and does not affect the determination of the peak value of NM. After obtaining the fatigue life of each asphalt mixture under different strain levels, the fatigue durability of asphalt mixtures was analyzed based on the relationship between fatigue life and strain level.

(9) $$NM=\frac{S_{i}\times N_{i}}{S_{0}\times N_{0}}$$

where *S*_0_ is the initial stiffness modulus of asphalt mixtures, and the value at the 50th loading is taken as the initial value in this research (with *N*_0_ correspondingly set to 50), MPa; *S_i_* is the stiffness modulus of asphalt mixtures when the number of loading cycles is *N_i_*, MPa.

## 3. Results and Discussions

### 3.1. Material Properties of RGAs

**Figure 2:**
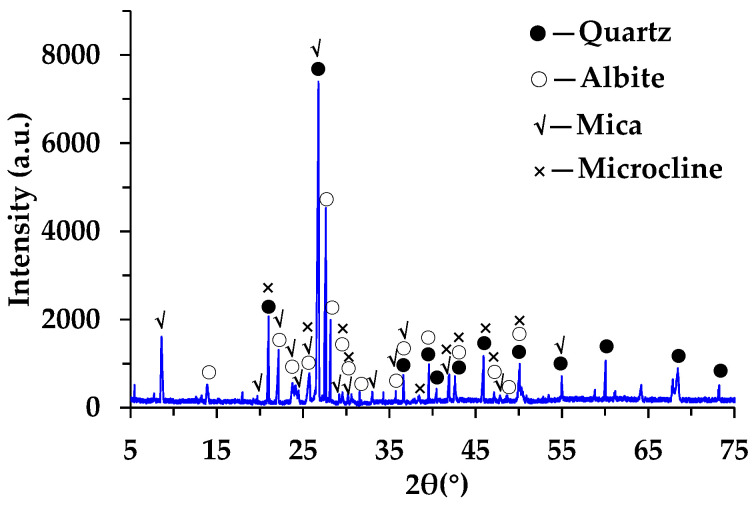
Mineral phases of RGA provided by XRD.

**Figure 3:**
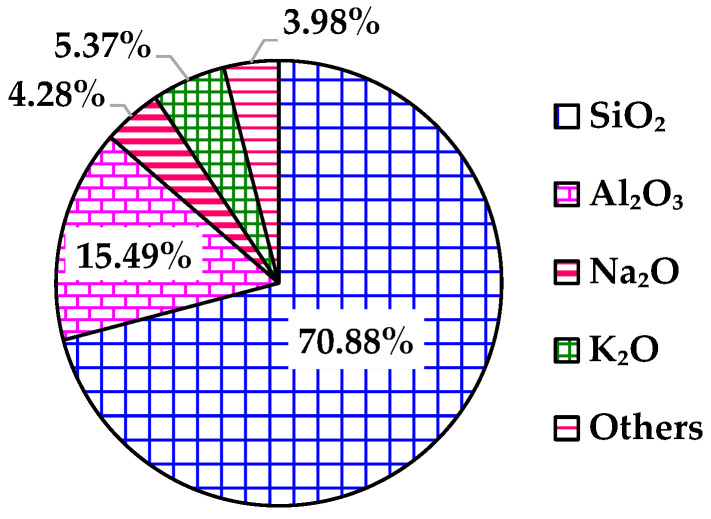
Chemical composition of RGAs provided by XRF.

The XRD results of RGAs are presented in Figure 2. The diffraction pattern shows that there are many diffraction peaks of different intensities, indicating that the mineral types of RGAs are rich. Based on the matching degree of the main diffraction peaks, it is concluded that the main minerals in RGAs are quartz, mica (muscovite and biotite), albite, and microcline. According to the form of elemental oxides, these minerals are mainly composed of SiO_2_, Al_2_O_3_, Na_2_O and K_2_O, which is consistent with the analysis results of XRF. As shown in Figure 3, the total content of the four main chemical components exceeds 96%, with SiO_2_ alone reaching 70.88%. Groome and Hall proposed distinguishing the acidity or alkalinity of rocks based on the content of SiO_2_ [28]. Rocks containing over 65% of SiO_2_ are classified as acidic rocks. It is evident that RGAs are acidic aggregates. The bonding performance between acidic aggregates and asphalt, which also exhibits weak acidity, is poor. Therefore, reducing this adverse effect of acidic aggregates is a key prerequisite for their utilization in asphalt mixtures.

The key difference between the RGA preparation process adopted in this study and the traditional crushed-stone preparation process lies in the integration of multiple cleaning treatments. This design is mainly motivated by the geological complexities of the tunnel-crossing areas such as faults, which cause the generated tunnel-muck raw material to occasionally contain excessive mud and impurities. Therefore, testing the mud content of RGAs is a critical prerequisite for determining whether the proposed RGA-processing method is effective and reliable. One batch of RGCAs is randomly selected for testing mud content, and the results of six parallel tests for RGCAs with different particle-size ranges are presented in Table 4. It can be seen that the mud content shows an upward trend as the particle-size range of RGCA decreases. Nevertheless, according to the Chinese technical specifications [29], the mud content of coarse aggregates (content of particles with a size smaller than 0.075 mm) should be less than 1%, and the mud content of RGCAs in all three particle-size ranges meets the requirements of technical specifications. However, the mud content of RGCA of 3–5 mm is quite close to the specified upper limit. Therefore, it is necessary to statistically determine whether there is still a significant difference between them. The *t*-test results show that the *t*_statistic_ is smaller than −*t*_(0.05, 5)_ (see Table 4), so the null hypothesis (H_0_) should be rejected, and the alternative hypothesis (H_1_) is accepted. Mud content test results and *t*-test results confirm that the integration of multi-cleaning technology in the production process of RGAs is successful.

**Figure 4:**
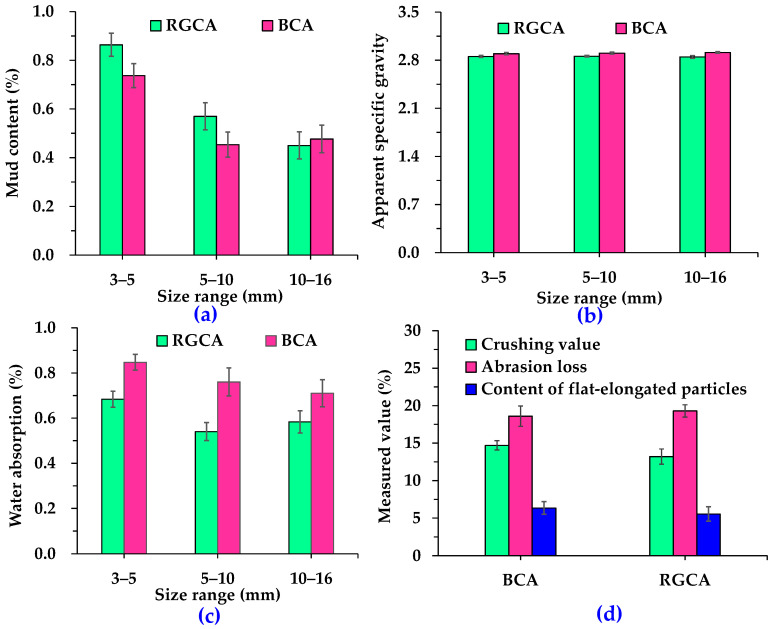
Comparison of conventional technical indexes between RGCA and BCA: (**a**) mud content; (**b**) apparent specific gravity; (**c**) water absorption; (**d**) crushing value, abrasion loss, and content of flat-elongated particles.

To further analyze the homogeneity and stability of RGA properties during large-scale production using the process illustrated in Figure 1, one batch of RGAs was randomly selected as the starting point, followed by selecting one batch every 10 days thereafter. Specifically, samples were taken from a total of three chosen batches to test their conventional technical properties. Figure 4 displays the main conventional technical indexes of RGCAs and basalt coarse aggregates (BCAs). According to the requirement of Chinese technical specifications [29], the mud content, water absorption, crushing value, Los Angeles abrasion loss, and content of flat-elongated particles of coarse aggregates for surface-layer asphalt mixtures should not exceed 1%, 2%, 26%, 28% and 12%, respectively, while the apparent specific gravity needs to be more than 2.6. Clearly, all the conventional technical indexes of RGCAs and BCAs meet the requirements of technical specifications. In detail, as the particle size decreases, the difference in mud content between RGCAs and BCAs increases, with RGCAs having a higher mud content. It indicates that it is highly necessary to clean tunnel muck using multi-cleaning technology; otherwise, the mud content of RGCAs is likely to exceed the specification limits. The apparent specific gravities of RGCAs and BCAs do not differ much. RGCAs with particle-size ranges of 3–5 mm, 5–10 mm, and 10–16 mm are 1.42%, 1.58% and 2.20% lighter, respectively, than BCAs with the same particle-size ranges. The water-absorption values of RGCAs and BCAs are 0.54–0.68% and 0.71–0.85%, respectively. Results suggest that the water absorption of BCAs is slightly higher, which may be related to the porous structure of basalt. Compared with BCAs, RGCAs exhibit a 10.2% lower crushing value, a 3.8% higher abrasion loss, and a 12.6% lower content of flat-elongated particles. This indicates that RGCAs possess better resistance to impact damage and comparable wear resistance to BCAs. These features provide advantages for the utilization of RGCAs in anti-skid asphalt surface.

**Figure 5:**
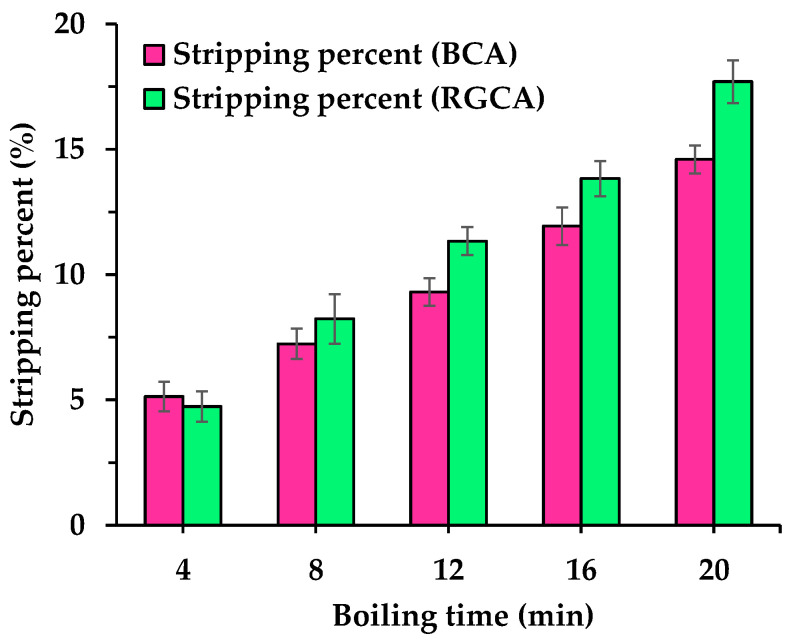
Test results of bonding performance between aggregates and asphalt.

Test results of the bonding performance between coarse aggregates and SBS-modified asphalt under boiling water damage conditions are shown in Figure 5. It shows that the stripping percent of SBS-modified asphalt from the surfaces of RGCAs and BCAs gradually increases for both as boiling time extends. In terms of standard deviation, although the test results fluctuate within a certain range, they still exhibit a relatively obvious trend of change. In the early stage of water damage, the difference in asphalt-stripping percent between the two types of aggregates is small; however, as boiling time increases, that of RGCAs becomes higher. After 20 min of boiling-water damage, the asphalt-stripping percent corresponding to RGCAs and BCAs is 17.7% and 14.6%, respectively, with that of RGCAs being 21.2% higher than that of BCAs. This demonstrates that RGCAs impair the bonding performance between aggregates and asphalt, even when high-grade SBS-modified asphalt is used. Therefore, it is necessary to improve the bonding performance between RGAs and asphalt to facilitate its utilization in surface-layer asphalt mixtures.

### 3.2. Material Properties of RGA-Basalt Hybrid Aggregates

**Figure 6:**
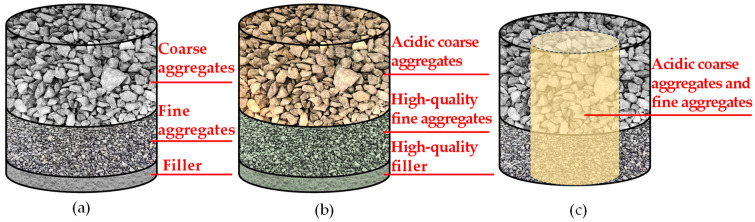
Aggregate composition of different asphalt mixtures: (**a**) asphalt mixture composed of conventional aggregates; (**b**) asphalt mixture containing acidic coarse aggregates; (**c**) asphalt mixture containing both acidic coarse and fine aggregates.

The raw mineral materials in the asphalt mixture are coarse aggregates, fine aggregates, and filler, as shown in Figure 6a. According to previous research about the application of acidic aggregates in asphalt mixtures, in order to mitigate their adverse effects on the performance of asphalt mixtures, acidic aggregates are commonly blended with high-quality aggregates to form hybrid aggregates. Existing research predominantly employs acidic aggregates as the coarse fraction, blended with high-quality fine aggregates or fillers [9,21,22,23], as shown in Figure 6b. For example, Zhao et al. prepared an asphalt mixture with granite coarse aggregates and steel slag fine aggregates (SSFA) [9], while Chen et al. applied SSFA and steel slag powder (SSP) filler to enhance the performance of mixtures containing gneiss coarse aggregates [21]. Despite these efforts, the approaches fail to achieve the simultaneous utilization of acidic coarse and fine aggregates in asphalt mixtures. This is because the fine aggregates contain a considerable amount of powder; if the fine aggregates are completely acidic aggregates, the acidic powder would significantly influence the function of the asphalt mastic. However, during the preparation process of aggregates, coarse aggregates and fine aggregates are produced simultaneously. This selective and prioritized utilization of acidic coarse aggregates results in resource waste and makes the utilization of fine aggregates more difficult. A method for the collaborative utilization of acidic coarse aggregates and fine aggregates is urgently needed.

In this research, a new method to realize the collaborative utilization of RGCAs and RGFAs was proposed. As shown in Figure 6c, it involves simultaneously blending RGCAs and RGFAs with high-quality BCAs and basalt fine aggregates (BFAs) to form hybrid aggregates, balancing the performance of asphalt mixtures by reducing the volume proportion of RGAs in hybrid aggregates. In China, when constructing asphalt pavements in areas with a severe water-damage environment, the used aggregates exhibit poor bonding performance with asphalt, so high-alkaline fillers such as cement are usually used to improve the performance of asphalt mixtures, especially for the surface-layer asphalt mixtures. To promote the recycling of RGAs in actual pavement surfaces in mountainous areas, this study also adopts this approach to enhance the bonding performance between RGAs and asphalt. Therefore, suitable RGA volume proportions in hybrid aggregates were discussed in this study for both scenarios of before and after cement reinforcement. In the experiment, four types of hybrid aggregates with RGA volume proportions of 40%, 50%, 60%, and 70%, respectively, were included.

**Figure 7:**
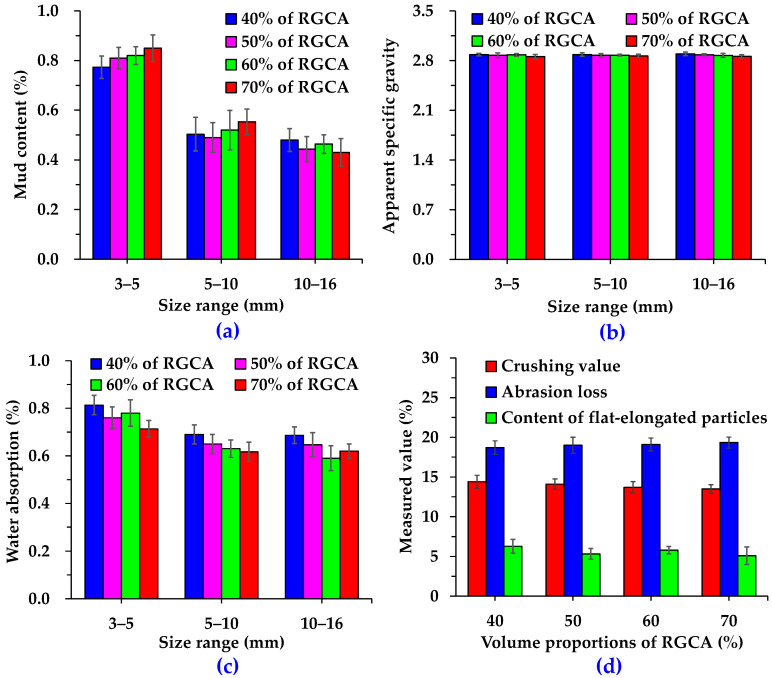
Main conventional technical indexes of hybrid coarse aggregates: (**a**) mud content; (**b**) apparent specific gravity; (**c**) water absorption; (**d**) crushing value, abrasion loss, and content of flat-elongated particles.

**Figure 8:**
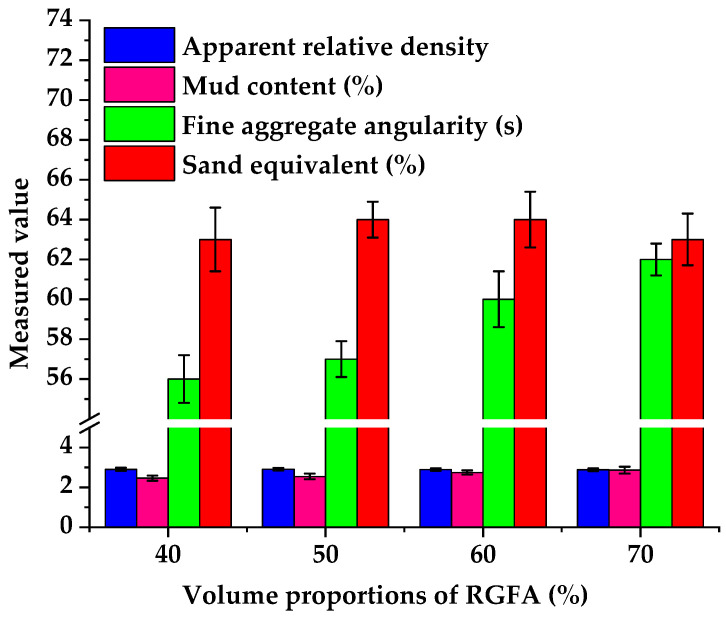
Main conventional technical indexes of hybrid fine aggregates.

The main conventional technical indexes of hybrid aggregates composed of RGAs and basalt aggregates are presented in Figure 7 and Figure 8. Figure 7 shows that the volume proportion of RGCAs does not obviously influence the technical properties of hybrid coarse aggregates. Specifically, for hybrid coarse aggregates with RGCA volume proportions of 40%, 50%, 60%, and 70%, respectively, the mud content is 0.43–0.85%, the apparent specific gravity is 2.857–2.895, the water absorption is 0.59–0.81%, the crushing value is 13.5–14.4%, the Los Angeles abrasion is 18.7–19.3%, and the content of flat-elongated particles is 5.1–6.3%. Results suggest that the main conventional technical indexes of the hybrid coarse aggregates with different RGCA volume proportions meet the requirements of Chinese technical specifications [29].

In terms of fine aggregates for surface-layer asphalt mixtures, the Chinese technical specifications require their apparent specific gravity, angularity, and sand equivalent to be more than 2.50, 30 s, and 60%, respectively, while the mud content should not exceed 3% [29]. Figure 8 shows that the main technical indexes of hybrid fine aggregates with RGFA volume proportions of 40%, 50%, 60%, and 70%, respectively, also meet the requirements of technical specifications [29]. In detail, the apparent specific gravity is 2.889–2.911, the mud content is 2.47–2.87%, the angularity is 56–62%, and the sand equivalent is 63–64%. The angularity of hybrid fine aggregates increases significantly with the increase in RGFA volume proportion. This is likely due to RGFAs altering the interlocking skeleton and contact states of particles in the hybrid fine aggregates.

### 3.3. Design Results of Asphalt Mixtures Incorporating Hybrid Aggregates

**Figure 9:**
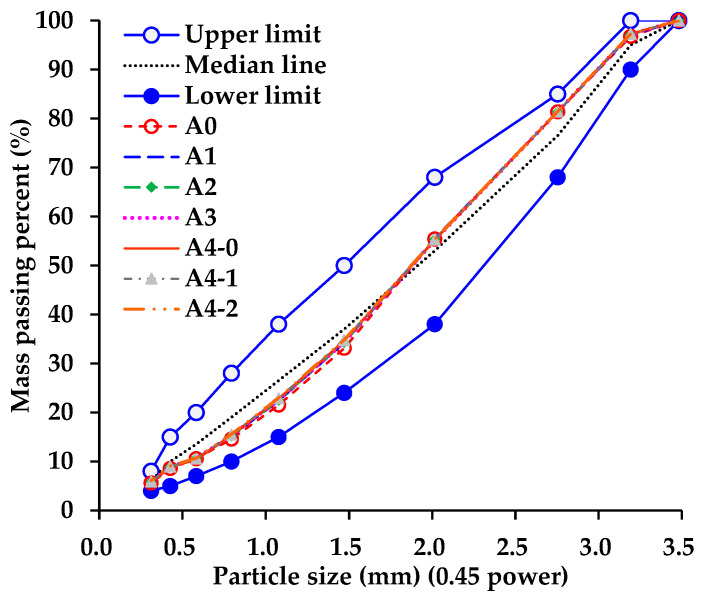
Hybrid gradations used in this research.

The suitable volume proportion of coarse aggregates with size ranges of 10–16 mm, 5–10 mm, and 3–5 mm, including fine aggregate (0–3 mm) and filler, was determined to be 20%, 26%, 18%, 32%, and 4%, respectively, based on the control group of A0 (pure basalt aggregate-based asphalt mixture). Aggregate types and volume proportions of each asphalt mixture are shown in Table 5, and the corresponding hybrid gradation curves are shown in Figure 9. As shown in Table 5, RGAs account for 40%, 50%, 60%, and 70% of the total aggregate volume in A1, A2, A3, and A4 (A4-0, A4-1, A4-2), respectively, and cement accounts for 25% and 50% of the total filler volume in A4-1 and A4-2, respectively. Figure 1 shows that, in the coordinate system with mass passing percent as the vertical axis, the hybrid gradations of these asphalt mixtures show little difference. This suggests that, although RGAs and basalt aggregates differ in density, the disparity is insufficient to markedly alter the hybrid gradations of asphalt mixtures. Furthermore, although cement and limestone powder exhibit notable differences in density, their limited dosage as fillers also exerts little effect on the hybrid gradations of asphalt mixtures. The design results of the volumetric properties of asphalt mixtures are presented in Table 6. This shows that all the main volumetric indexes of each asphalt mixture meet the design requirements. In detail, the number of voids in the mineral aggregates (VMAs) of asphalt mixtures incorporating RGAs is slightly larger than that of A0, indicating that RGAs have slightly altered the interlocking and stacking state of the aggregate skeleton. A higher VMA value means more asphalt is required to fill the gaps in the aggregate skeleton. Therefore, the optimum asphalt content (OAC) and voids filled with asphalt (VFA) of each asphalt mixture incorporating RGAs are also slightly larger than those of A0.

### 3.4. Main Performance of Asphalt Mixtures Incorporating Hybrid Aggregates

#### 3.4.1. Moisture-Induced Damage Resistance

**Figure 10:**
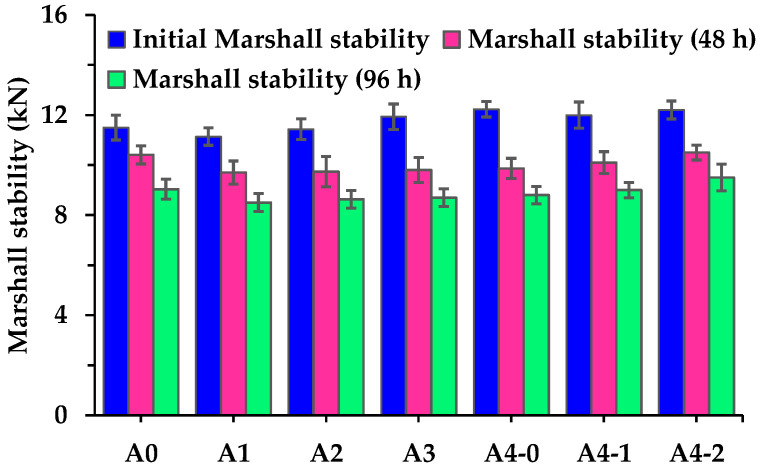
Marshall stabilities of asphalt mixtures before and after hot water damage.

The Marshall stabilities of asphalt mixtures before and after hot water damage (in a 60 °C water bath) are shown in Figure 10. The introduction of RGAs clearly affects the initial Marshall stabilities of asphalt mixtures. Compared with control group (A0), the initial Marshall stability of A1 decreases by 3.2%. It suggests that a low RGA volume proportion (40%) slightly weakens the initial Marshall stability of asphalt mixtures. However, as the RGA volume proportion increases, this trend reverses, and the Marshall stability of asphalt mixtures continues to rise. When the RGA volume proportion reaches 70%, the Marshall stability rises to 12.2 kN (A4-0). Although high-alkaline cement has been proven to improve the bonding properties of the asphalt-aggregate system, its contribution to improving the Marshall stability of asphalt mixtures in this research is limited. It indicates that the improvement in the Marshall stability of asphalt mixtures with high RGA proportions may mainly be attributed to RGAs optimizing the interlocking skeleton structure and contact state of aggregate particles. After hot water damage, the Marshall stability of each asphalt mixture decreases to varying degrees. The decrease grows more pronounced as the hot water damage duration prolongs. This indicates that the Marshall stability of asphalt mixtures is sensitive to hot water damage.

**Figure 11:**
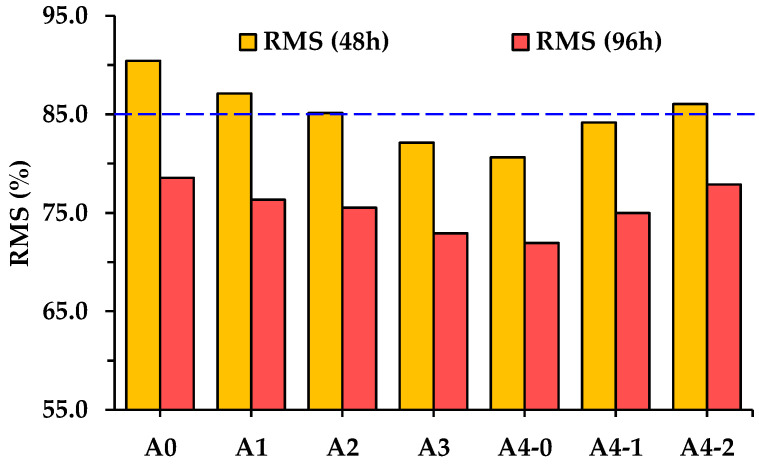
RMS of asphalt mixtures after hot water damage with different durations.

Figure 11 shows that the Marshall stability of specimens after hot water damage is also sensitive to the RGA volume proportion, and as the proportion increases, the Marshall stability decreases more significantly. Specifically, after 48 h or 96 h of hot water damage, the Marshall stability of A4-0 always decreases the most, with the RMS being only 80.7% or 71.9%, respectively. The RMS begins to rise when cement is used as a partial filler. When the cement amount reaches 50% of the total filler volume, the RMS after 48 h and 96 h of hot water damage recovers to 86.1% and 77.9%, respectively. It proves that cement plays a positive role in improving the hot-water-damage resistance of asphalt mixtures incorporating acid aggregates. Even after long-term hot water damage, the RMS of A4-2 is equivalent to that of A0. In China, the RMS of asphalt mixtures prepared with modified asphalt is required to be no less than 85% after 48 h of hot water damage [29]. For asphalt mixtures with hybrid aggregates, the RMS of A1, A2, and A4-2 meets this requirement, while that of A2 is near the lower limit.

**Figure 12:**
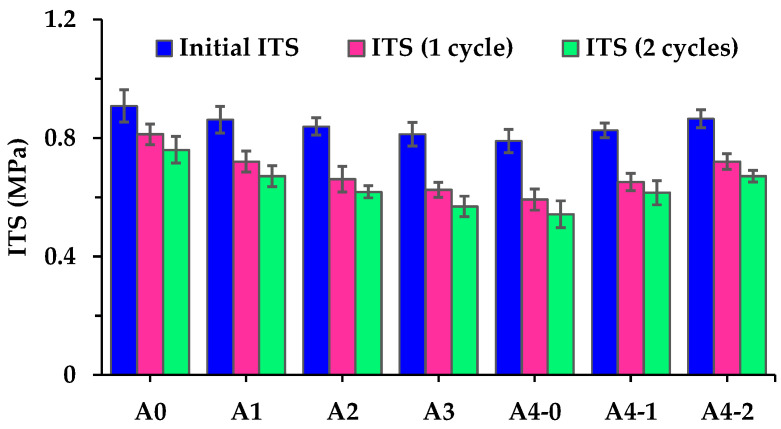
ITS values of asphalt mixtures before and after freeze–thaw damage.

The ITS values of asphalt mixtures before and after freeze–thaw damage are shown in Figure 12. It shows that the effect of RGAs on the initial ITS of asphalt mixtures is more pronounced. RGAs reduce initial ITS of asphalt mixtures to varying degrees, and as the RGA volume proportion increases, the initial ITS continues to decrease. When the RGA volume proportion reaches 70%, the initial ITS of A4-0 is 13.0% lower than that of A0. It suggests that, unlike what happens in Marshall stability test, the bonding performance of the asphalt–aggregate system exerts a more significant influence on ITS than the interlocking skeleton structure and contact state of aggregate particles. The changes in initial ITS caused by cement also confirm it. When the cement amount reaches 50% of the total filler volume, the initial ITS of asphalt mixture (A4-2) is 9.6% larger than that of A4-0. This enhancement is attributed to the improved bonding performance of the asphalt–aggregate system facilitated by cement.

**Figure 13:**
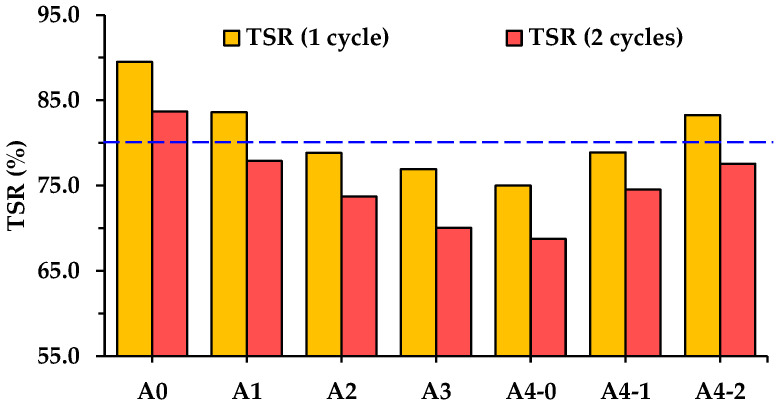
TSR of asphalt mixtures after different cycles of freeze–thaw damage.

Figure 12 also shows that the ITS of asphalt mixture is quite sensitive to the freeze–thaw damage. The ITS of each asphalt mixture decreases after freeze–thaw damage, and asphalt mixtures with high RGA volume proportions exhibit a more pronounced reduce in ITS. As shown in Figure 13, the ITS loss percent of asphalt mixture incorporating 70% RGA by volume of total aggregates (A4-0) reaches 25.0% and 31.2% after freeze–thaw damage for 1 and 2 cycles, respectively. Cement also plays a positive role in maintaining the ITS of asphalt mixtures during freeze–thaw cycle damage, as it significantly improves the TSR of asphalt mixtures incorporating RGAs. When the cement amount reaches 50% of the total filler volume, the TSR of asphalt mixtures reaches 83.3% and 77.6% after freeze–thaw damage for 1 and 2 cycles, respectively. However, the TSR of A4-2 after freeze–thaw damage for 2 cycles is still lower than that of A0. It further indicates that cyclic freeze–thaw process causes severe damage to the ITS of asphalt mixtures. In China, the TSR of asphalt mixture prepared with modified asphalt is required to be no less than 80% after freeze–thaw damage for 1 cycle [29]. Among asphalt mixtures with hybrid aggregates, the TSR of A1 and A4-2 meets this requirement. After comprehensively considering RMS and TSR, in terms of moisture-induced damage resistance, if only the hybrid aggregate approach is adopted, the suitable RGA volume proportion in hybrid aggregates should not exceed 40%. If cement is additionally used as a reinforcing material, the RGA volume proportion can be increased to 70%. It is recommended to use RGAs, following the mix designs of A1 and A4-2. Therefore, the subsequent analysis will mainly compare the other aspects of engineering performance of A1 and A4-2 with those of A0.

#### 3.4.2. High-Temperature Deformation Resistance

**Figure 14:**
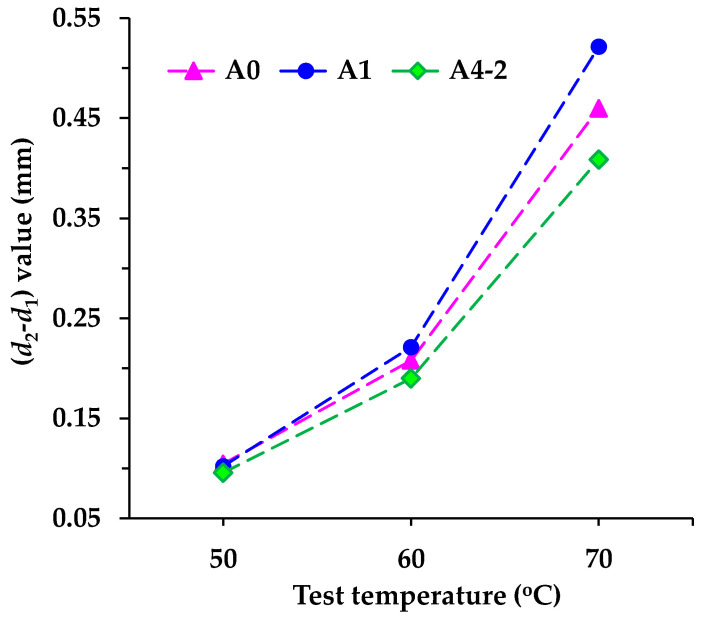
The (*d*_2_ − *d*_1_) values of asphalt mixtures obtained from a rutting test.

The average values of (*d*_2_ − *d*_1_) obtained from the rutting test are shown in Figure 14. Since the time interval, wheel pressure, and moving speed of the steel wheel are fixed for all asphalt mixture specimens, the value of (*d*_2_ − *d*_1_) as shown in Equation 4 reflects the difficulty of forming a rutting deformation in the asphalt mixture. The smaller the (*d*_2_ − *d*_1_) value, the more difficult it is to form a rutting deformation, indicating better high-temperature deformation resistance of asphalt mixture. Figure 14 shows that the (*d*_2_ − *d*_1_) value of each mixture increases sharply with rising temperature. It illustrates that higher temperatures obviously enhance the rheological behavior of asphalt mixtures and weaken their deformation resistance, consistent with the essential feature of asphalt as a temperature-sensitive material. Specifically, under a test environment of 50 °C, the (*d*_2_ − *d*_1_) values of the three asphalt mixtures differ slightly. As temperature increases, the difference becomes progressively pronounced. A1 exhibits the largest (*d*_2_ − *d*_1_) value, A4-2 the smallest, and A0 an intermediate value. Therefore, based on (*d*_2_ − *d*_1_) values, the high-temperature (60 °C or above) deformation resistance of the asphalt mixtures follows the order: A4-2 > A0 > A1.

**Figure 15:**
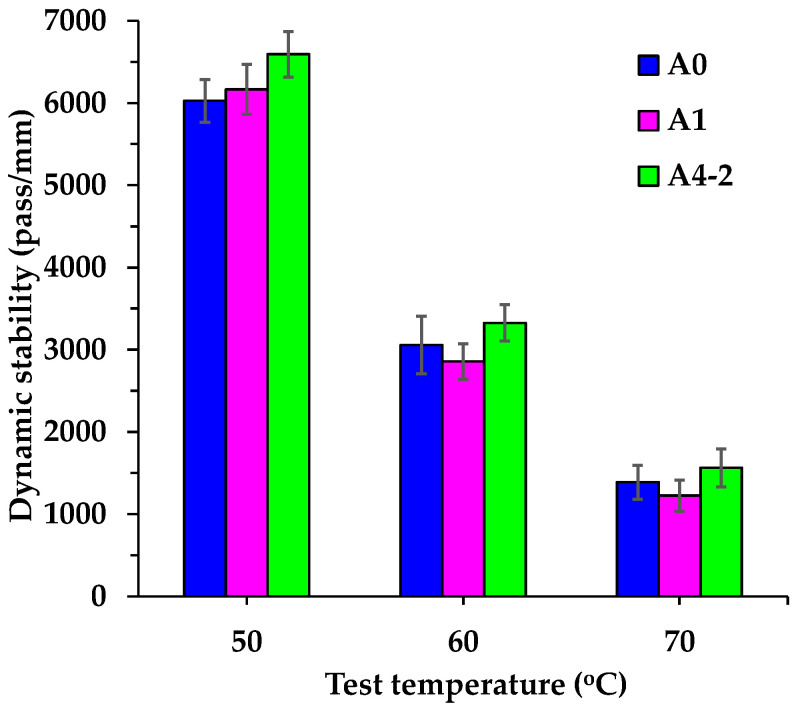
Dynamic stabilities of asphalt mixtures.

The dynamic stabilities shown in Figure 15 more intuitively demonstrate the deformation resistance of asphalt mixtures at different temperatures. The dynamic stability of all asphalt mixtures decreases significantly with rising temperature, meaning the number of wheel passes required to generate 1 mm of a rutting deformation is significantly reduced, making the asphalt mixtures more prone to rutting. Unlike the (*d*_2_ − *d*_1_) values at 50 °C in Figure 14, the dynamic stabilities measured at 50 °C clearly reflect the deformation resistance level of these three asphalt mixtures. This is mainly because the (*d*_2_ − *d*_1_) values of these three asphalt mixtures are inherently very small. When they serve as the denominator in the dynamic stability calculation equation, the minor differences in (*d*_2_ − *d*_1_) values among these three asphalt mixtures can cause significant variations in dynamic stability. As shown in Figure 15, in these circumstances, the dynamic stability of A1 and A4-2 is 2.3% and 9.4% higher than that of A0, respectively. It indicates that incorporating RGAs improves the deformation resistance of asphalt mixtures, and a high RGA volume proportion performs better. This may also be attributed to RGA optimizing the interlocking skeleton structure and contact state of aggregate particles. While the order of deformation resistance changes with rising temperature, at 60 °C or 70 °C, A4-2 consistently exhibits the highest dynamic stability, followed by A0, with A1 being the lowest. Compared with A0, the dynamic stability of A1 decreases by 6.5% (60 °C) and 11.6% (70 °C), respectively, while the dynamic stability of A4-2 increases by 8.8% (60 °C) and 12.6% (70 °C), respectively. It indicates that, in addition to the interlocking skeleton structure and contact state of aggregate particles, the bonding performance of asphalt mixtures also obviously affects the high-temperature stability, particularly when asphalt mixtures are subjected to 60 °C or above. Due to the poor bonding performance between acid RGAs and asphalt, the flow capability of A1 increases with rising temperature, resulting in relatively low dynamic stability at 60 °C and 70 °C. For A4-2, which incorporates both cement and a high RGA volume proportion, its high dynamic stability benefits from enhanced skeleton structure, stable particle contact, and improved aggregate–asphalt bonding performance. According to the strict requirements of Chinese technical specifications [29], modified asphalt mixtures used in summer hot zones must have a dynamic stability of no less than 2800 pass/mm when tested at 60 °C with a wheel pressure of 0.7 MPa. Although the dynamic stability of A1 at 60 °C is lower than that of A0 and A4-2, it still meets the requirements of technical specifications. However, in harsh high-temperature environments, A4-2 is recommended to ensure the asphalt pavement maintains excellent high-temperature deformation resistance.

#### 3.4.3. Low-Temperature Crack Resistance

**Figure 16:**
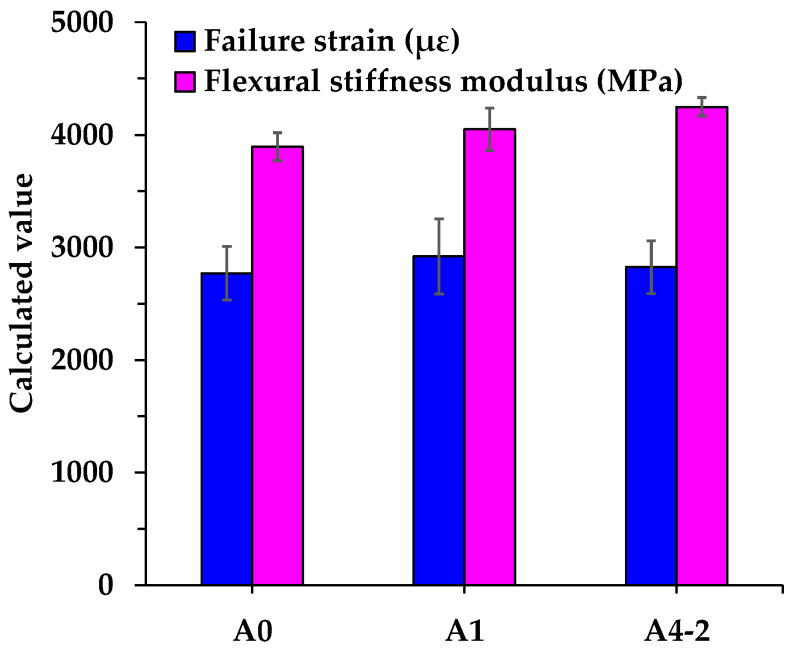
Failure strain and flexural stiffness modulus of asphalt mixtures.

**Figure 17:**
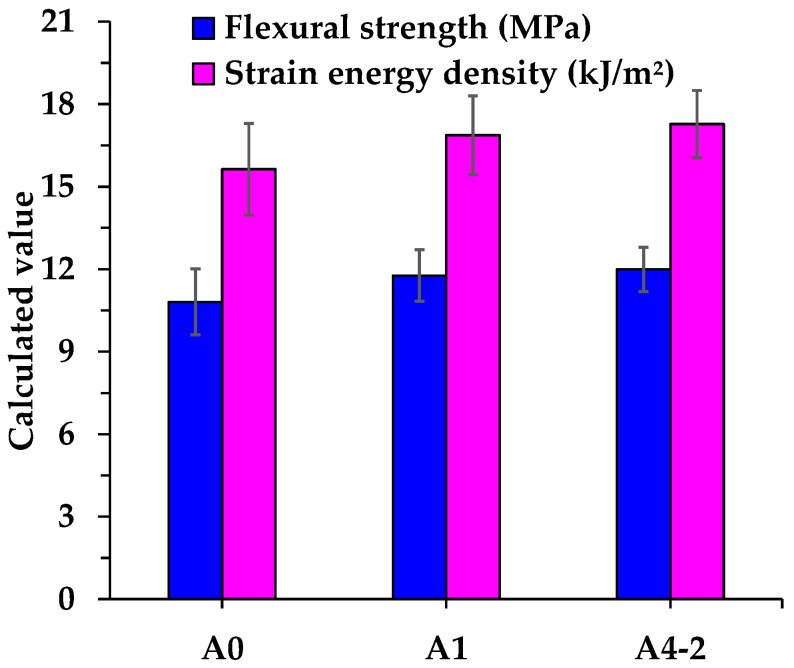
Flexural strength and strain energy density of asphalt mixtures.

The failure strain, flexural strength, flexural stiffness modulus, and strain energy density of asphalt mixtures are shown in Figure 16 and Figure 17. As shown in the figures, the introduction of RGAs and cement leads to varying degrees of improvement in the failure strain, flexural strength, and flexural stiffness modulus of asphalt mixtures. Compared with the control group (A0), the failure strain, flexural strength, and stiffness modulus of A1 increase by 5.4%, 8.9%, and 4.0%, respectively. For A4-2, the corresponding values rise by 2.0%, 10.9%, and 9.1%. It indicates that RGAs and cement not only enhance the low-temperature deformation capacity and bearing capacity of asphalt mixture, but also increase its rigidity, thereby elevating the risk of brittle cracking.

In China, the low-temperature crack resistance of asphalt mixtures is primarily determined by failure strain, which reflects the ability of asphalt mixtures to dissipate energy through deformation under load-induced damage. The low-temperature strain of asphalt mixtures is proven to be directly related to the properties of asphalt. A slight increase in failure strain of A1 and A4-2 may be mainly attributed to a small rise in the OAC, as A1 and A4-2 require more asphalt to fill the skeleton voids to obtain satisfactory asphalt mixtures. However, the failure strain of A4-2 is 3.2% lower than that of A1, implying that cement and RGA volume proportion also affect the low-temperature strain of asphalt mixtures. Relying solely on the single parameter of failure strain to assess low-temperature crack resistance of asphalt mixtures is insufficient. For example, although A4-2 exhibits a lower failure strain than A1, its flexural strength is approximately 1.9% higher, indicating a slight advantage in bearing capacity. Thus, the trends of failure strain and flexural strength of A4-2 are inconsistent when evaluating low-temperature crack resistance. Additionally, the strain of asphalt mixtures is highly sensitive to changes in composition, structure, and environmental factors related to asphalt mixtures. Even for the same asphalt mixture, significant fluctuations in failure strain may be observed in tests conducted at different times. In contrast, strain energy density integrates both strain and strength of asphalt mixtures, making it a more reasonable parameter for characterizing the low-temperature performance of asphalt mixtures. As shown in Figure 17, the strain energy density of A1 and A4-2 is 7.9% and 10.5% higher than that of A0, respectively. It suggests that the incorporation of RGA and cement improves the low-temperature crack resistance of asphalt mixtures, and the combination of RGA and cement yields even better performance.

#### 3.4.4. Fatigue Life and Fatigue Durability

**Figure 18:**
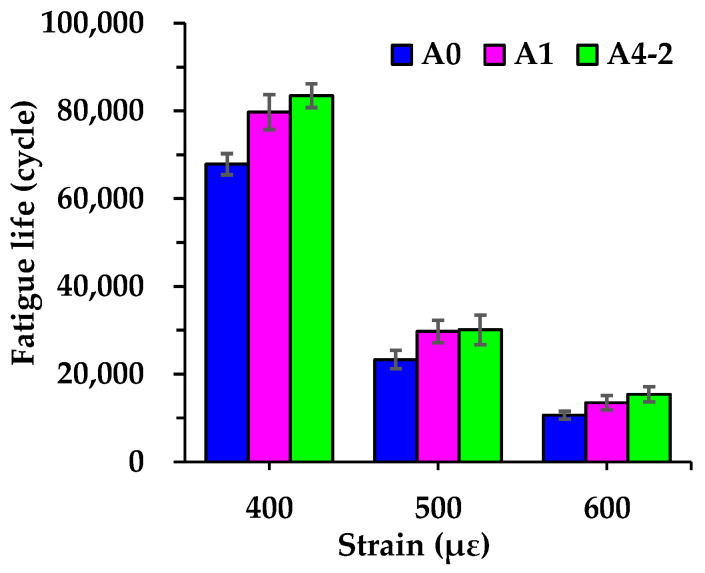
Fatigue life of asphalt mixtures.

Figure 18 displays the fatigue life of each asphalt mixture measured under different initial strain levels. It is evident that the fatigue life of all asphalt mixtures is sensitive to strain levels. As strain increases, the fatigue life decreases gradually. Specifically, when the strain increases from 400 με to 500 με, the fatigue life of A0, A1 and A4-2 decreases by 65.6%, 62.8%, and 63.9%, respectively. When the strain further increases from 500 με to 600 με, the corresponding decreases are 54.3%, 54.6%, and 48.8%, respectively. Among the three asphalt mixtures, although the difference in fatigue life between A1 and A4-2 at 500 με is not significant, under the same test conditions, A4-2 consistently exhibits the highest fatigue life, followed by A1, with A0 being the lowest. Compared with A0, the fatigue life of A1 and A4-2 is 17.5% and 23.0% higher at 400 με, 27.4% and 29.2% higher at 500 με, and 26.6% and 44.8% higher at 600 με, respectively. It illustrates that RGA alone or the combination of RGA and cement plays a positive role in improving the fatigue life of asphalt mixtures, with the synergy of high-volume RGA and cement showing a more significant improvement at high strain.

**Figure 19:**
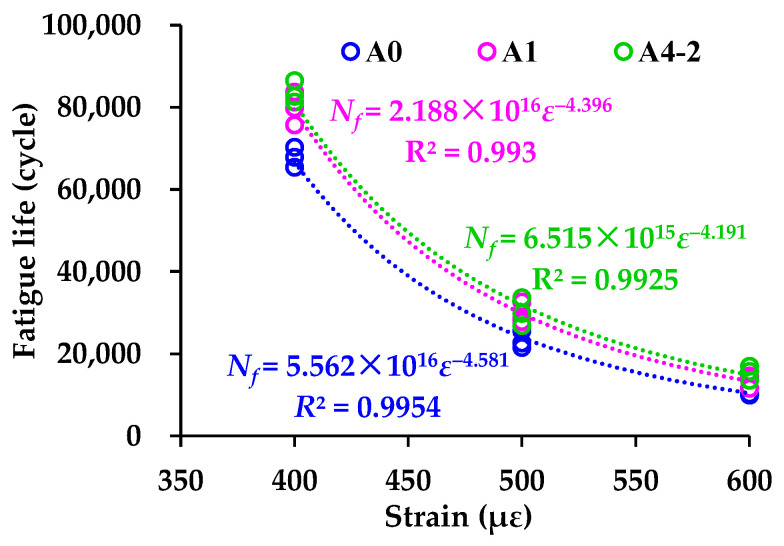
Fitting results of fatigue life in power function form.

As shown in Equation 10, the power function has been widely validated to be effectively describe the relationship between the fatigue life and stress or strain of asphalt mixtures. The parameter *b* reflects the sensitivity of *N_f_* to changes in *ε*, which characterizes the fatigue durability of asphalt mixtures. The fitting results of *N_f_* and *ε* using the power function are shown in Figure 19, which indicates that the *b*-values of A0, A1, and A4-2 are 4.581, 4.396, and 4.191, respectively. A lower *b*-value indicates the lower sensitivity of fatigue life to strain changes. Thus, RGAs alone or the combination of RGAs and cement also improves the fatigue durability of asphalt mixtures.

(10) $$N_{f}=a\varepsilon^{-b}$$

where *N_f_* is the fatigue life of asphalt mixtures, cycle; *ε* is the strain level, με; and *a* and *b* are constants related to the feature of asphalt mixtures.

Overall, after comprehensively weighing the performance of asphalt mixtures in various aspects, it can be concluded that blending RGA with basalt aggregates to form hybrid aggregates is an effective approach to recycling tunnel muck in surface-layer asphalt mixtures. When no other enhancement measures are applied, it is advisable to ensure the RGA volume proportion in hybrid aggregates does not exceed 40%. If a higher RGA recycling rate is required, it is recommended to use RGAs together with cement, maintaining RGA volume proportion in hybrid aggregates at 70% and the cement amount at 50% of the total filler volume. Both asphalt mixtures (A1 and A4-2) demonstrate performance comparable to that of pure basalt aggregate-based asphalt mixture (A0), with some performance even performing much better.

Compared with traditional schemes that depend entirely on combining acidic coarse aggregates with high-alkaline fine aggregates or fillers [9,21,22,23], the schemes proposed in this study offer distinct advantages. On one hand, they enable full-gradation utilization of both coarse and fine RGAs derived from tunnel muck. On the other hand, the scheme with a 40% RGA volume proportion in hybrid aggregates does not even require additional enhancement measures. In contrast, traditional schemes often require enhancement from high-alkaline fillers such as cement, steel slag powder [21], hydrated lime [22,23], etc.

### 3.5. Economic Benefits of Using RGAs in Asphalt Mixtures

The preparation cost of RGAs was quantified based on statistical data of resource consumption during actual production, mainly including labor costs, transportation costs, water and electricity costs, and equipment costs (rental, maintenance, and depreciation), etc., which were incurred during the stages of approximate selection of tunnel muck at the excavation site (S1), transportation to the temporary disposal site (S2), multiple cleaning treatments (S3), and four-stage crushing process (S4). In addition, if tunnel muck is not processed into aggregates for reuse, it must be transported outward to a dump for stockpiling or disposal. Processing tunnel muck into RGAs eliminates this disposal cost, which should be included as a cost saving when calculating the actual cost of RGAs. Table 7 summarizes the costs of each stage, cost savings, and the actual preparation cost of RGAs. Although the processing costs of coarse aggregates and fine aggregates differ, enterprises typically sell them at a unified price (regardless of particle size gradations). Thus, the cost analysis of RGAs does not distinguish between coarse and fine aggregates, and an average price is used instead. As shown in Table 7, the four-stage crushing process (S4) contributes the most to the total processing cost, accounting for 68.24%. This is because crushing requires specialized crushing, dust removal, and screening equipment, leading to high equipment and electricity consumption costs. The itemized processing costs of RGAs shown in Table 8 also support it. The equipment costs and the water and electricity costs account for 25.69% and 51.12% of the total (sum of S1 to S4), respectively. Labor costs account for 10.06%, which is relatively low. This is because part of the labor costs is categorized under other itemized costs; for example, the drivers’ salaries are classified as transportation costs.

In Hubei Province, China, basalt aggregates are mainly sourced from areas such as Jingshan City. A survey of aggregate-processing enterprises in Jingshan shows that the current ex-factory price of basalt aggregates ranges from 110 to 130 yuan/t, and the transportation cost is 0.47 to 0.53 yuan/(t·km). For the economic benefit analysis of replacing basalt aggregates with RGA in surface-layer asphalt mixtures, the ex-factory price of basalt aggregates and the transportation cost (for both RGAs and basalt) were set at 120 yuan/t and 0.5 yuan/(t·km), respectively. Assuming the transportation distances of RGAs and basalt aggregates to the mixing plant are *S_g_* and *S_b_*, respectively, the costs of one ton of RGAs (*P_g_*) and basalt aggregates (*P_b_*) delivered to the same mixing plant are shown in Equation 11 and Equation 12, respectively. It can be inferred that with the same aggregate procurement budget, RGAs can meet the needs of engineering projects located farther from the supply source. When the difference in transportation distance between RGAs and basalt aggregates does not exceed 161.48 km, the unit weight cost of RGAs delivered to the mixing plant is lower.

(11) $$P_{g}=0.5S_{g}+39.26$$

(12) $$P_{b}=0.5S_{b}+120$$

A case study was conducted to analyze the economic benefits of using locally sourced RGAs to pave a 1 km-long, 4 cm-thick, single-lane-wide asphalt pavement surface layer. The aggregate costs of the optimal RGA utilization schemes (A1 and A4-2) were calculated under four different transportation distance of external basalt aggregates to the mixing plant (50 km, 100 km, 150 km, and 200 km), with RGAs transported locally at a fixed distance of 20 km from its processing site to the mixing plant. Table 9 lists the aggregate amount required for A0, A1, and A4-2, and Table 10 shows the corresponding aggregate costs. Results show that the aggregate costs of all three mixtures increase significantly with the extension of basalt transportation distance. This is attributed to the high dependence of A0 on basalt aggregates and the direct correlation between logistics costs and transportation distance. Meanwhile, under the same basalt transportation distance, the aggregate cost of A0 is the highest, followed by A1, with A4-2 being the lowest. When the basalt transportation distance ranges from 50 km to 200 km, A1 achieves an aggregate cost reduction of 26.56–31.19% compared with A0, while A4-2 realizes a more substantial reduction of 46.49–54.56%. It confirms that blending RGAs with basalt aggregates and maintaining RGA volume proportion in the hybrid aggregates at 40% or 70% yields significant economic benefits in terms of aggregate costs. However, practical engineering applications require consideration of cost changes from other raw materials. For example, different asphalt mixtures demand varying asphalt dosages (e.g., RGA-incorporated mixtures typically require slightly higher OAC to fill skeleton voids), and the introduction of cement in A4-2 will also adjust the overall raw material costs. These factors should be incorporated into comprehensive economic evaluations to ensure the feasibility of RGA utilization schemes in actual projects.

## 4. Conclusions

In this research, to realize the full-gradation utilization of tunnel muck-derived recycled granite aggregates (RGAs), the feasibility of simultaneously utilizing recycled granite coarse aggregates (RGCAs) and fine aggregates (RGFAs) in surface-layer asphalt mixtures via hybrid with basalt was fully discussed. Some main conclusions are as follows:

(1) The multi-cleaning technology, consisting of a vibrating grid steel mesh, a water-flushing device, and a vibrating feeder, can effectively separate stone blocks from impurities, mud, and debris in tunnel muck. The mud content of prepared RGCAs is no more than 1%. Compared with BCAs, RGCAs show better impact damage resistance and comparable wear resistance.
(2) RGAs are mainly composed of quartz, mica (muscovite and biotite), albite, and microcline. These minerals contribute to a SiO_2_ content of up to 70.88% in RGAs, making RGAs acidic aggregates. Water boiling test results confirm that it is necessary to improve the bonding performance between RGAs and asphalt.
(3) The main conventional technical indexes of RGA–basalt hybrid aggregates with various RGA volume proportions of 40%, 50%, 60%, and 70% meet the requirements of Chinese technical specifications. Mud content ranges from 0.43% to 0.85% for RGCAs and 2.47% to 2.87% for RGFAs.
(4) The VMAs of asphalt mixtures incorporating RGAs is slightly larger than that of pure basalt aggregate-based asphalt mixtures, indicating that RGAs modify the interlocking skeleton and contact states of aggregate particles. A higher VMA value also results in larger OAC and VFA.
(5) Blending RGAs with basalt to form hybrid aggregates is an effective way to realize full-gradation utilization of tunnel muck-derived RGA in surface-layer asphalt mixtures. Without additional enhancement measures, the RGA volume proportion in hybrid aggregates should be no more than 40% (A1 scheme). With cement accounting for 50% of the total filler volume, the RGA volume proportion can be increased to 70% (A4-2 scheme).
(6) Applying the two schemes (A1 and A4-2) to paving a 1 km-long, 4 cm-thick, single-lane-wide asphalt pavement surface layer with locally sourced RGAs, when external basalt aggregates are transported over 50–200 km, these schemes yield at least 26.56% aggregate cost savings.

This study confirms that partially incorporating RGAs into basalt to form hybrid aggregates serves as an effective approach to achieving the full-gradation utilization of RGAs sourced from tunnel muck. Future work requires two key efforts. It is necessary to explore the potential application of RGAs in other pavement layers to expand its utilization scenarios. It is also essential to integrate RGA use with practical engineering, specifically evaluating its performance in real-world projects, and assessing the construction costs and ecological benefits of such applications.

## Figures and Tables

**Table 1:** Conventional technical properties of fillers.

Measured Index		Limestone Powder	Cement	Requirements
Density (g/cm^3^)		2.717	3.115	≥2.5
Appearance		Light gray	Dark gray	——
Percent passing (%)	0.6 mm	100	100	100
	0.15 mm	93.5	96.6	90–100
	0.075 mm	85.1	89.9	75–100

**Table 2:** Conventional technical properties of SBS-modified asphalt.

Measured Index	Results	Requirements
Ductility (5 °C, 5 cm/min; cm)	39	≥20
Softening point (°C)	77.3	≥60
Penetration (25 °C, 100 g, 5 s; 0.1 mm)	58	40–60
Viscosity (135 °C; Pa·s)	1.0	≤3
Solubility (%)	99.3	≥99
Elasticity resume (25 °C; %)	77	≥75

**Table 3:** Sizes of specimens and main test conditions.

Performance	Sizes of Specimens	Main Test Conditions
Moisture-induced damage resistance	Marshall specimens Diameter: 100 mm Thickness: 63.5 mm	Hot water (60 °C) damage: 48, 96 h Freeze–thaw damage: 1, 2 cycles Loading rate: 50 mm/min
High-temperature deformation resistance	Plate specimens Length: 300 mm Width: 300 mm Thickness: 50 mm	Test temperature: 50, 60 and 70 °C Wheel pressure: 0.7 MPa wheel speed: 42 pass/min Test duration: 1 h per test
Low-temperature crack resistance	Beam specimens Length: 250 mm Width: 30 mm Thickness: 35 mm	Test temperature: −10 °C Deformation speed: 50 mm/min
Fatigue performance	Beam specimens Length: 380 mm Width: 63.5 mm Thickness: 50 mm	Test temperature: 15 °C Loading frequency: 10 Hz Strain control mode

**Table 4:** Mud content test results and *t*-test results.

Particle Size Range (mm)	Test Results (%)						Average Value (%)	Standard Deviation
	Sample 1	Sample 2	Sample 3	Sample 4	Sample 5	Sample 6		
10–16	0.35	0.43	0.44	0.38	0.48	0.51	0.43	0.0598
5–10	0.55	0.57	0.49	0.61	0.65	0.47	0.56	0.0689
3–5	0.93	0.87	0.95	0.79	0.85	0.90	0.88	0.0581
*t*-test	H_0_	H_1_	Free degree	Significance level		*t*_statistic_	*t*_(0.05, 5)_	Decision
	*μ ≥* 1	*μ <* 1	5	0.05		−4.988	2.015	Reject H_0_

**Table 5:** Aggregate types and volume proportions of each asphalt mixture.

Mixture Type	Coarse Aggregate (%)						Fine Aggregate (%)		Filler (%)	
	10–16 mm		5–10 mm		3–5 mm		0–3 mm			
	BCA	RGCA	BCA	RGCA	BCA	RGCA	BFA	RGFA	Limestone Powder	Cement
A0	20	0	26	0	18	0	32	0	4	0
A1	12	8	15.6	10.4	10.8	7.2	19.2	12.8	4	0
A2	10	10	13	13	9	9	16	16	4	0
A3	8	12	10.4	15.6	7.2	10.8	12.8	19.2	4	0
A4-0	6	14	7.8	18.2	5.4	12.6	9.6	22.4	4	0
A4-1	6	14	7.8	18.2	5.4	12.6	9.6	22.4	3	1
A4-2	6	14	7.8	18.2	5.4	12.6	9.6	22.4	2	2

**Table 6:** Main volumetric indexes of designed asphalt mixtures.

Volumetric Index	A0	A1	A2	A3	A4-0	A4-1	A4-2	Requirements
Air voids (%)	4.0	4.0	4.0	4.0	4.0	4.0	4.0	4.0
OAC (%)	4.75	4.85	4.90	4.90	5.00	5.00	5.00	—
VMA (%)	15.2	15.4	15.5	15.5	15.6	15.6	15.6	≥14
VFA (%)	73.7	74.0	74.2	74.2	74.4	74.4	74.4	65–75

**Table 7:** Costs of each stage, cost savings, and actual preparation cost of RGA.

Costs of Each Stage (yuan/t)				Cost Savings (yuan/t)	Actual Cost (yuan/t)
S1	S2	S3	S4		
4.28	7.00	6.08	37.30	15.40	39.26

**Table 8:** Itemized processing costs of RGA.

Subitem	Equipment	Water and Electricity	Transportation	Labor
Costs (yuan/t)	14.04	27.94	7.10	5.50
Percent (%)	25.69	51.12	12.99	10.06

**Table 9:** Aggregate amount required for A0, A1 and A4-2.

Mixture Type	Aggregate Type	Aggregate Amount (t)				Total Amount (t)
		10–16 mm	5–10 mm	3–5 mm	0–3 mm	
A0	Basalt	72.55	93.92	64.68	118.48	349.63
A1	RGA	28.91	37.71	26.08	46.97	139.67
	Basalt	43.44	56.23	38.72	70.93	209.32
A4-2	RGA	50.42	65.78	45.49	81.92	243.61
	Basalt	21.65	28.02	19.30	35.35	104.32

**Table 10:** Aggregate costs of A0, A1 and A4-2.

Transportation Distance of Basalt Aggregates (km)	Aggregate Costs (yuan)		
	A0	A1	A4-2
50	50,696	37,232	27,127
100	59,437	42,465	29,735
150	68,178	47,698	32,343
200	76,919	52,931	34,951

## Data Availability

The original contributions presented in this study are included in the article. Further inquiries can be directed to the corresponding author.
